# Whole-transcriptome profiling and identification of cold tolerance-related ceRNA networks in *japonica* rice varieties

**DOI:** 10.3389/fpls.2024.1260591

**Published:** 2024-03-19

**Authors:** Hao Wang, Yan Jia, Xu Bai, Jin Wang, Ge Liu, Haixing Wang, Yulong Wu, Junying Xin, Huimiao Ma, Zhenyu Liu, Detang Zou, Hongwei Zhao

**Affiliations:** ^1^ Key Laboratory of Germplasm Enhancement, Physiology and Ecology of Food Crops in Cold Region, Ministry of Education, Northeast Agricultural University, Harbin, China; ^2^ Bei Da Huang Kenfeng Seed Limited Company, Research and Breeding Center, Harbin, China

**Keywords:** rice, low-temperature, competing endogenous RNA, mRNA, miRNA

## Abstract

**Introduction:**

Low-temperature stress negatively impacts rice yield, posing a significant risk to food security. While previous studies have explored the physiological and linear gene expression alterations in rice under low-temperature conditions, the changes in competing endogenous RNA (ceRNA) networks remain largely unexamined.

**Methods:**

We conducted RNA sequencing on two *japonica* rice varieties with differing cold-tolerance capabilities to establish ceRNA networks. This enabled us to investigate the transcriptional regulatory network and molecular mechanisms that rice employs in response to low-temperature stress.

**Results:**

We identified 364 differentially expressed circular RNAs (circRNAs), 224 differentially expressed microRNAs (miRNAs), and 12,183 differentially expressed messenger RNAs (mRNAs). *WRKY* family was the most prominent transcription factor family involved in cold tolerance. Based on the expression patterns and targeted relationships of these differentially expressed RNAs, we discerned five potential ceRNA networks related to low-temperature stress in rice: osa-miR166j-5p from the miR166 family was associated with cold tolerance; osa-miR528-3p and osa-miR156j-3p were linked to stress response; and osa-miR156j-3p was involved in the antioxidant system. In addition, *Os03g0152000* in the antioxidant system, as well as *Os12g0491800* and *Os05g0381400*, correlated with the corresponding stress response and circRNAs in the network. A gene sequence difference analysis and phenotypic validation of *Os11g0685700* (*OsWRKY61*) within the *WRKY* family suggested its potential role in regulating cold tolerance in rice.

**Discussion and conclusion:**

We identified *Os11g0685700* (*OsWRKY61*) as a promising candidate gene for enhancing cold tolerance in *japonica* rice. The candidate miRNAs, mRNAs, and circRNAs uncovered in this study are valuable targets for researchers and breeders. Our findings will facilitate the development of cold-tolerant rice varieties from multiple angles and provide critical directions for future research into the functions of cold-tolerance-related miRNAs, mRNAs, and circRNAs in rice.

## 1 Introduction

Heilongjiang Province in China is a major hub for the production of high-quality *japonica* rice (Song et al., 2020). Cold damage is a global issue that particularly affects rice-producing countries like Japan, Korea, and China. The problem is exacerbated by the high latitude and low temperature in Heilongjiang Province, where rice seedlings are growing in April, with temperatures ranging from 3 to 15°C. Rice crops prefer warm conditions (Liu et al., 2020a; Liu et al., 2022). The capacity of rice to withstand cold temperatures is commonly referred to as cold tolerance or cold resistance. Typically, rice thrives at temperatures between 15 and 33°C. When temperature drops below this range, it can disrupt the plant’s water metabolic balance by reducing its water-absorption capacity and transpiration rates, eventually causing cellular water loss. Low temperatures also reduce the enzymatic activity of superoxide dismutase (SOD), catalase (CAT), and peroxidase (POD) in rice. This results in a sharp rise in levels of reactive oxygen species (ROS), which subsequently leads to lipid peroxidation in cell membranes, oxidative protein degradation, nucleic acid damage, and enzyme inactivation, thereby triggering programmed cell death (Wang et al., 2021; Xu et al., 2022b). During the seed germination phase, low temperatures can substantially reduce germination rates. In the seedling stage, they can cause symptoms such as leaf curling, seedling stiffness, wilting, reduced dry matter weight, stunted growth, and even plant death in extreme cases. These conditions can also adversely affect the late tillering stage of the plant’s life cycle (Shimono, 2011). A significant yield loss or even complete crop failure can occur in case of early snowfall (Jia et al., 2019). Furthermore, low temperatures during the reproductive growth stage can cause pollen sterility and a substantial reduction in fruit set. It is estimated that cold damage reduces China’s annual rice production by 3 to 5 billion kg. The vulnerability of Heilongjiang to cold inversion during spring seasons poses a significant risk to the growth and development of rice seedlings in the region.

circRNAs are non-coding RNA molecules that form a closed loop consisting of one or more exons connected by a spliceosome complex (Xu et al., 2022a). Unlike typical linear RNAs, circRNAs lack a 5’ cap structure and 3’ polyadenylation, making them resistant to nucleic acid exonucleases. As a result, they are relatively stable and conserved (Li et al., 2016). A single gene can generate multiple types of circRNAs, some of which are expressed at higher levels than their corresponding linear RNAs and function as miRNA sponges (Wu et al., 2020). Previously, circRNAs were believed to be the byproducts of splicing errors until the 1970s, when Sanger et al. identified these closed-loop RNA molecules in plant viruses and confirmed their presence in eukaryotic cells (Sanger et al., 1976). The advent of high-throughput sequencing technology allowed the identification of plant circRNAs in *Arabidopsis thaliana* in 2014 (Zhao et al., 2019). Similar to their animal counterparts, plants produce diverse types of circRNAs through selective back-splicing (Romero-Barrios et al., 2018). circRNA-related studies in a variety of plants, including maize (Han et al., 2020), rice (Lu et al., 2015), wheat (Xu et al., 2019), barley (Darbani et al., 2016), and soybean (Wang et al., 2020) have revealed that circRNAs can originate from exons, introns, or intergenic regions (Liu et al., 2020b). They have also shown that circRNA expression patterns often vary across different tissues and developmental stages (Huang et al., 2020). Furthermore, evidence suggests that circRNAs are more stable than linear RNAs (Zhong et al., 2018) and may play roles in various plant functions through specialized mechanisms, including regulating chlorophyll metabolism, hormone signaling, flower development, fruit ripening, and leaf senescence (Zhang et al., 2017). Additionally, they modulate distinct responses to different stress conditions.

circRNAs can serve as ceRNA or miRNA sponges, playing a pivotal role in various biological processes. A study identified 31 differentially expressed circRNAs, 47 differentially expressed miRNAs, and 4,779 differentially expressed mRNAs in rice. Using Cytoscape software, the researchers constructed miRNA-mediated regulatory and ceRNA networks. Their findings indicated that the circRNAs A02:23507399|23531438 are post-transcriptionally significant and regulate anther development (Liang et al., 2019). Additionally, senescence-associated circRNAs are implicated in flag leaf senescence via parental gene expression and ceRNA network regulation (Huang et al., 2021). However, the literature is scant concerning the role of circRNAs in regulating low-temperature responses in rice. To date, none of the studies have explored ceRNA networks specifically related to cold tolerance in rice. Consequently, it remains uncertain whether circRNA–miRNA–mRNA targeting interaction networks participate in rice’s response to low-temperature stress.

The objective of this study was to construct a ceRNA network to understand how rice responds to low-temperature stress. Our findings shed light on the functional roles of *japonica* rice and lay the groundwork for future research into circRNAs, as well as offer insights into the regulatory mechanisms governing cold tolerance in this rice variety.

## 2 Materials and methods

### 2.1 Plant materials and cold treatments

The study was carried out in November 2021 at Northeast Agricultural University in Harbin, Heilongjiang Province, China (longitude: 126°22’-126°50’; latitude: 45°34’-45°46’N). Two rice varieties were selected for the experiment: JL (Jilin Sunset), which is cold-tolerant, and JH (Jinhe), which has low cold tolerance. For seed preparation, rice seeds were sequentially sterilized using a 75% ethanol solution for 2 min and a 2% H2O2 solution for 30 min. The seeds were then rinsed three times with sterile water and allowed to germinate for 3 days. Subsequently, one germinated seed was placed in each hole of a hydroponic setup. The nutrient solution for the hydroponic system was formulated based on the International Rice Research Institute’s (IRRI) standard nutrient solution guidelines. The seedlings were incubated in an intelligent artificial climate chamber (model FYS-20, Nanjing, China) under controlled conditions that practically simulated the seedling growth conditions of Heilongjiang rice: 20°C day/18°C night temperature, 12-h light/12-h dark cycle, and 50% relative humidity. When the seedlings reached the “three leaves and one heart” developmental stage, they were transferred to the same climate chamber but set at 4°C for cold treatment (Huang et al., 2021).

### 2.2 Sample collection and mRNA extraction for sequencing and quality control

Leaves from the JL and JH rice varieties were harvested at 0, 4, 12, 24, and 48 h following the initiation of cold treatment. Each time point had three replicates for each variety. Each replicate was composed of a pooled sample of leaves from three individual seedlings, resulting in a total of 30 samples collected across both varieties (2 varieties × 5 time points × 3 replicates). These samples were immediately frozen in liquid nitrogen and stored at -80°C until further analysis. Total RNA was extracted from the leaf samples using an RNA extraction kit (Tiangen Biotech, Beijing, China). The concentration and integrity of the RNA was assessed using a NanoDrop 2000 spectrophotometer (Thermo Fisher Scientific, Waltham, MA, USA). Residual rRNA was removed from the total RNA using an rRNA Removal Kit (Epicentre Technologies, Madison, WI, USA). An aliquot of the purified RNA underwent first-strand cDNA synthesis, followed by second-strand cDNA synthesis, end repair, and 3’ end addition.

After sequencing, the raw data were subjected to quality analysis, and low-quality sequences and junctions were removed. The remaining sequences were aligned to the Nipponbare reference genome (*Oryza sativa*/IRGSP_1.0_release_62) using the default parameter of HISAT2 (v2.2.1.0) (Kim et al., 2015), which was made available through the Ensemble Plants database (https://plants.ensembl.org/index.html). Post-alignment, StringTie (version 2.2.0) is used to assemble and quantify the sequence according to the default parameters (Pertea et al., 2016). Raw gene expression levels were determined based on read counts for each gene and were subsequently normalized using Fragments Per Kilobase Million (FPKM). Differential gene expression analysis between sample groups was conducted using DESeq2 (version 1.28). Genes were considered differentially expressed if they met the following criteria: an absolute log_2_ fold-change (|log_2_^FC^|) greater than or equal to 1 and a false discovery rate (FDR) < 0.01. The FDR was calculated by adjusting the p-value for the significance of differences between groups.

### 2.3 Sequencing and differential analysis of circRNA

An aliquot of the extracted RNA was treated with RNaseR (Epicentre, Madison, WI, USA) to selectively remove linear RNA and construct a circRNA-seq library. This library was sequenced by Biomarker Ltd. (Beijing, China) on the Illumina NovaSeq 6000 platform. Low-quality reads, defined as those with > 5% unknown (‘N’) bases, splice sequences, or > 50% bases with a low-quality score (Q ≤ 20), were excluded from the raw data. The remaining high-quality reads were aligned to the rice reference genome using the HISAT software (Kim et al., 2015). circRNAs were identified using the Find_circ software (version1.1) with default parameters (Memczak et al., 2013). The expression levels of the identified circRNAs were normalized following a previously established method (Zhu et al., 2019). Differential expression analysis was conducted using DESeq software, with a screening criterion for differentially expressed circRNAs set at an absolute log_2_ fold change (|log_2_^FC^|) > 1 and P-values < 0.05. Genes giving rise to differentially expressed circRNAs were subjected to Gene Ontology (GO) (Version2.10.0) enrichment analysis (Consortium, 2004). GO terms with P < 0.05 were considered significantly enriched. Additionally, Kyoto Gene and Genome Encyclopedia (KEGG) R package (version1.4.0) pathway enrichment analysis was performed using the R package (Kanehisa et al., 2016); pathways with a P < 0.05 were deemed significantly enriched.

### 2.4 miRNA library construction, sequencing, and quality control

Total RNA was isolated from each sample using TRIzol reagent (Invitrogen, USA) and miRNA was extracted from the total RNA through size fractionation on 15% PAGE gels. RNA molecules ranging from 18 to 30 nucleotide (nt) in length were purified from these gels. Adapters were ligated to both the 5′ and 3′ ends of the miRNA molecules, followed by reverse transcription and PCR amplification, resulting in the creation of 30 miRNA libraries. These libraries were then sequenced on an Illumina HiSeq 2500 platform.

Upon sequencing, the raw reads underwent initial quality control steps. This involved the removal of sequences < 18 nt or > 30 nt, sequences with unidentified bases making up ≥ 10% of the read, and sequences lacking 3′ end splice sites. The 3′ end splice sequences were subsequently trimmed to generate clean reads, which were then aligned against various databases, including Silva (Version138) (Quast et al., 2012), GtRNAdb (version 3.0) (Chan and Lowe, 2016), Repbase (version21.01) (Jurka et al., 2005), and Rfam (version12.0) (Nawrocki et al., 2015). Following this, unannotated reads containing miRNAs were identified by filtering out snoRNA, tRNA, snRNA, rRNA, and other non-coding RNAs (ncRNAs), as well as repetitive sequences. These filtered, unannotated reads were then aligned to the rice reference genome (*Oryza sativa*, MSU_v7.0) using Bowtie software (version 1.1.2) (Langmead and Salzberg, 2012) with default parameters to examine their expression and distribution. Differential expression analysis was subsequently conducted using DESeq2 (version 1.28), with screening criteria set at an absolute log-fold change (|log_1.5_^FC^|) of ≥ 1 and P-values ≤ 0.05.

### 2.5 Analysis of the ceRNA network construction

To construct the circRNA-miRNA-mRNA network, we initially selected differentially expressed circRNAs, differentially expressed miRNAs, and differentially expressed mRNAs in accordance with ceRNA theory. TargetFinder (Version 2.0) (Bo and Wang, 2005) was employed to predict the target interactions between circRNAs and miRNAs, while psRNATarget (Version2017) (Dai and Zhao, 2011) was used for predicting the target interactions between miRNAs and mRNAs. We list the detailed parameters used by both software. TargetFinder: with parameters as -c (Prediction score cutoff value) 4 were used to predict target genes. Mismatches, single-nucleotide gaps or single-nucleotide bulges are assessed with a penalty of +1. G:U base pairs are assessed a penalty of +0.5. Penalty scores are doubled at positions 2-13 relative to the 5’ end of the small RNA query sequence. Duplexes are rejected if they: have more than one single-nucleotide bulge or gap; have more than seven total mismatches, G:U base pairs, bulges and gaps; have more than four total mismatches or four total G:U base pairs. PsRNAtarget:Max Expectation cutoff: 5.0; HSP length for scoring: 19; Penalty for GU pair: 0.5; Penalty for other mismatch: 1.0; Penalty for opening gap: 2.0 Penalty for extending gap: 0.5. RNAhybrid is used to calculate RNA : RNA interaction regions with the default parameters, according to the thermodynamics of RNA structure formation and several “targeted mimicry rules” (Krüger and Rehmsmeier, 2006); psRobot was used to analyze the targeting relationship between circRNA:miRNA and miRNA:mRNA and the correlation between them according to the default parameters (Wu et al., 2012). A p-value < 0.05 was used to select statistically significant RNA : RNA and target:ncRNA interactions predicted with the four programs, in addition to: a “maximum expectation score” of 5 for psRNATarget, a “maximum prediction score” of 6.5 for TargetFinder, a low minimum free energy (MFE) value for RNAhydrid, and “low target scores” between a threshold of 0 and 5 for psRobot. These relationships were further refined based on the correlations between their expression levels. Finally, ceRNA networks relevant to cold tolerance regulation were visualized using Cytoscape software (Version3.2.1) (Shannon et al., 2003).

### 2.6 RNA extraction and qRT-PCR

Rice leaves were subjected to cold treatment at 4°C for 0, 4, 12, 24, and 48 h. Total RNA was extracted using the TranZol-UpRNA kit (Tiangen Biotechnology, Beijing, China). The reverse transcription process was conducted using the HiFiScript cDNA synthesis kit (Cwbio, Beijing, China). qRT-PCR was carried out on a Roche LightCycler 2.10 system, with each sample having three technical replicates and three biological replicates. The housekeeping gene *Actin1* was used for expression normalization. Relative gene expression levels were calculated using the 2^-ΔΔCt^ method. The correlation between RNA-Seq and qRT-PCR data was assessed using Pearson’s correlation, based on absolute log-fold change (|log_10_^FC^|), and was analyzed using SPSS software (SPSS Inc., Chicago, IL, USA). The primer sequences used for the PCR reactions are listed in **Supplementary Table 1**.

### 2.7 Sequencing and sequence alignment analysis of *OsWRKY61*

The coding sequence (CDS) region of the *OsWRKY61* gene was cloned and sequenced from both the JH and JL rice varieties using PCR. Sequence alignment was conducted using DNAMAN software (version 5.0), with the genes from the Nipponbare genome serving as a reference.

### 2.8 Construction of *OsWRKY61* overexpression plants

For *OsWRKY61* overexpression, cDNA from the JH rice variety was used as a template. The full-length *OsWRKY61* coding sequence was cloned into the pUbi:1390-3Flag plant expression vector, which was subsequently transformed into *Agrobacterium tumefaciens* strain EHA105. For the rice transformation, mature rice seeds were dehusked and sterilized in a 3% sodium hypochlorite solution for 30 min. The seeds were then rinsed three times with sterile distilled water and placed on MS basal medium supplemented with 500 mg/L proline, 300 mg/L casein hydrolysate, 2 mg/L 2,4-dichlorophenoxyacetic acid (2,4-D), and 30 g/L sucrose. Finally, the seeds were incubated for one week. Freshly induced calli were inoculated with the prepared Agrobacterium culture and transferred to NB medium composed of N6 macro-elements and B5 micro-elements, along with 500 mg/L proline, 300 mg/L casein hydrolysate, and 30 g/L sucrose. After two days in these conditions (minimal to no light exposure, 28°C), the calli were transferred to fresh NB medium containing 2 mg/L 2,4-D, 50 mg/L hygromycin, and 500 mg/L cefotaxime. Subsequently, resistant calli were subcultured on fresh plates at two-week intervals over a period of four weeks. These were then transferred to MS medium supplemented with 0.2 mg/L α-naphthalene acetic acid (NAA), 3 mg/L 6-benzylaminopurine (6-BA), and 30 mg/L hygromycin until shoots regenerated. For the final rooting and further growth, shoots were transferred to 1/2 MS medium containing 0.5 mg/L NAA. Eventually, the plantlets were transferred to a greenhouse for further growth.

### 2.9 Determination of cold tolerance in *OsWRKY61* overexpression plants

The overexpression and wild-type (WT) plants that were previously screened were cultivated together in the “Three leaves and one heart” stage under the following conditions: 20°C day/18°C night temperature, 12-h light/12-h dark cycle, and 50% relative humidity. These plants then underwent cold treatment at 4°C and subsequently samples were collected at 0, 12, 24, and 48 h following the initiation of cold treatment. Commercial kits from Suzhou Grace Biotechnology Co., Ltd. (Jiangsu, China) were employed to quantify the levels of SOD, POD, PRO, and MDA, in accordance with the manufacturer’s guidelines.

## 3 Results

### 3.1 High-throughput sequencing of mRNA and circRNA

To comprehensively assess the expression profiles of circRNAs and mRNAs related to cold tolerance in *japonica* rice seedlings, we performed whole-transcriptome RNA-seq analyses. These analyses targeted the leaves of control and cold-treated seedlings from two *japonica* rice varieties, JH and JL, using an Illumina sequencer. Each sample set, consisting of two varieties at five time points with three replicates each, was analyzed in three independent biological replicates. After filtering out low-quality and contaminated reads, the aggregated data from both JH and JL yielded approximately 3.3 billion high-quality reads. These high-quality reads mapped to between 95.97% and 97.86% of the rice reference genome. Furthermore, all samples displayed Q30 values above 93.64% and their GC contents ranged from 46.55% to 48.21% (**Supplementary Table 2**), thereby confirming the reliability of the RNA-seq data. Our results generated a robust dataset for the subsequent examination of mRNA and circRNA expression profiles.

### 3.2 mRNA identification and differential analysis

**Figure 1:**
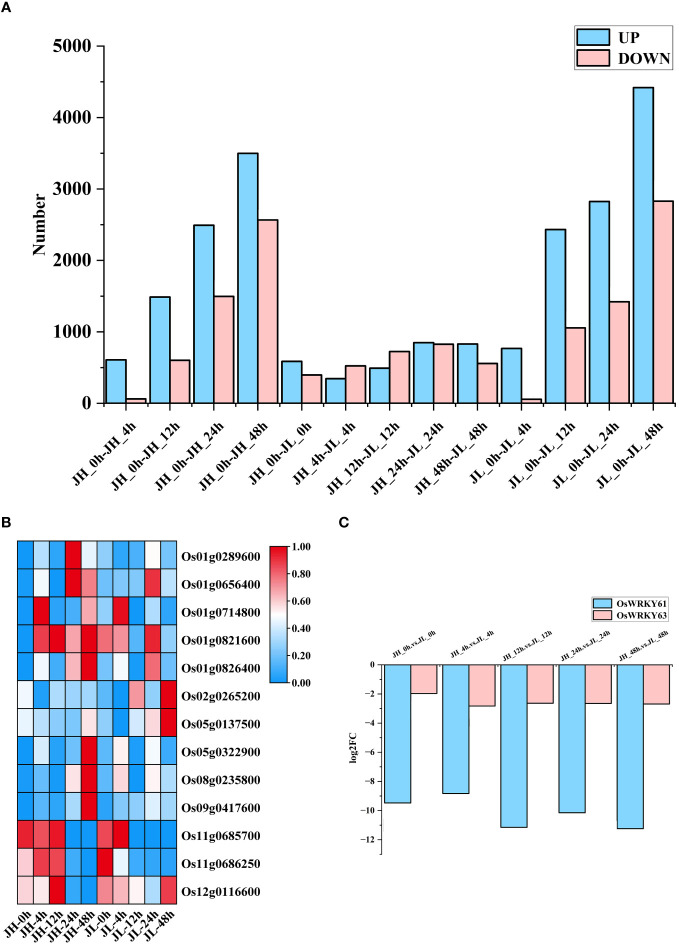
Differential analysis of mRNA expression in rice seedling leaves. **(A)** Number of differentially expressed genes in JH and JL varieties at various time points following cold treatment. **(B)** Representation of WRKY family members among differentially expressed transcription factors. **(C)** Temporal expression patterns of *OsWRKY61* and *OsWRKY63* based on transcriptome data after cold treatment.

Clean reads from each sample were aligned to the reference genome, yielding 36,850 mRNAs. Based on the screening criteria of an absolute |log_2_^FC^| of ≥ 1 and FDR < 0.01, we identified 12,817 and 15,806 differentially expressed mRNAs in the JH and JL varieties, respectively. Compared to the control at 0 h, JH exhibited 8,088 significantly upregulated genes and 4,729 significantly downregulated genes, whereas JL showed 10,446 upregulated and 5,363 downregulated genes. A comparative analysis between the two varieties at identical time points revealed 3,102 genes significantly upregulated and 3,028 genes downregulated in JL compared to JH (**Figure 1A**). Genomic coordinates and expression values of all differential mRNAs are organized in **Supplementary Table 3**. Our interspecies comparison identified a considerable number of differential transcription factors, with the *WRKY* family containing the highest count at 13 (**Figure 1B**), and notably, previous research has demonstrated the importance of WRKY in various abiotic stress response pathways, including those for drought, salinity, temperature, and ultraviolet radiation (Li et al., 2020).

### 3.3 Functionality of *OsWRKY63* in cold tolerance

The gene *OsWRKY63* has been determined to play a role in cold regulation (Zhang et al., 2022). Under normal conditions, the overexpression strain of *Os11g0686250* (*OsWRKY63*) from the *WRKY* family displayed no significant differences compared to the wild type. However, under cold stress conditions, the overexpression strain showed both a significantly lower fruit set rate and reduced seedling survival rate compared to the wild type. In contrast, CRISPR/Cas9-mediated knockdown of *OsWRKY63* led to enhanced cold tolerance (Zhang et al., 2022). Importantly, among the differentially expressed *WRKY* transcription factors identified, *Os11g0685700* (*OsWRKY61*) exhibited a similar response pattern compared to *OsWRKY63*. It showed high expression exclusively in the JH variety across all five time points, while its expression in the JL variety was virtually negligible, displaying a substantial fold change at each time point. Further, our cold-response sequencing data indicated that *OsWRKY61* had a higher ploidy level compared to *OsWRKY63* (**Figure 1C**). Based on these findings, we hypothesize that *OsWRKY61* likely possesses a regulatory function similar to that of *OsWRKY63* in rice, and we have initiated experiments involving its overexpression.

### 3.4 Identification and differential analysis of circRNA

**Figure 2:**
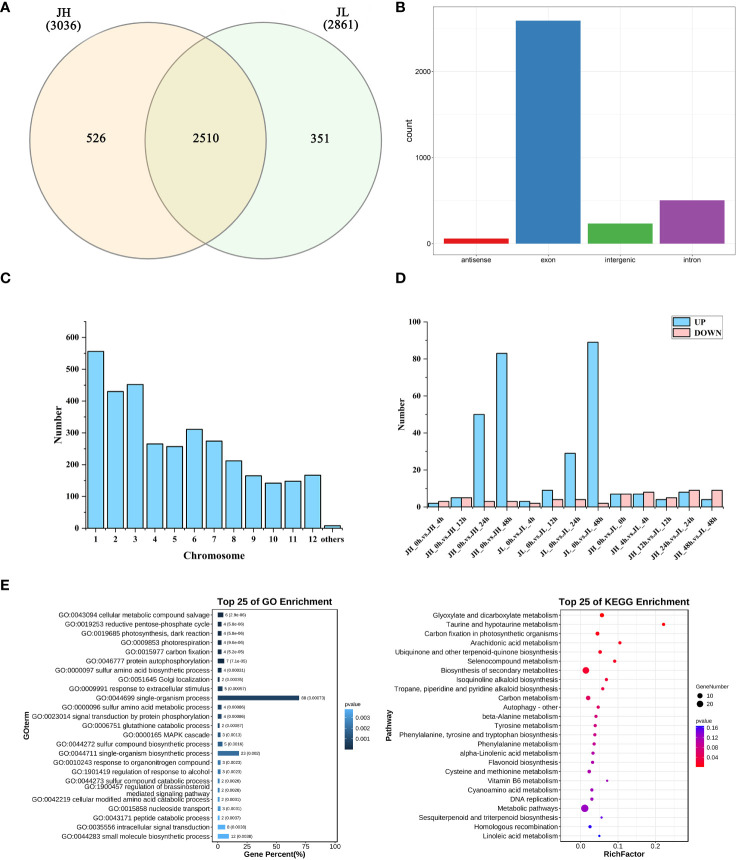
Identification and differential analysis of circRNAs in rice seedling leaves. **(A)** Count of differentially expressed genes in both JH and JL varieties after cold treatment. **(B)** Categorization of circRNAs based on their genomic position. **(C)** Chromosomal distribution of identified circRNAs. **(D)** Number of differentially expressed genes in JH and JL at each time point following cold treatment. **(E)** GO and KEGG enrichment analyses of parental genes for all differentially expressed circRNAs.

A comprehensive set of 3,387 circRNAs was identified from the transcriptome data of 30 samples (**Supplementary Table 4**), using CIRI2 (version1.2) with default parameters, a circRNA prediction software. The expression levels of these circRNAs, measured in FPKM pair values, predominantly ranged from -1 to 2 (**Supplementary Figure 1**). These identified circRNAs were categorized into three distinct groups based on their expression in the rice varieties: 526 circRNAs were unique to JH, 351 were exclusive to JL, and 2,510 were expressed in both varieties (**Figure 2A**). Further classification based on genomic location showed that 234 circRNAs were in intergenic regions, 503 in intronic regions, 2,591 in exonic regions, and 59 in antisense strands of genes (**Figure 2B**). Chromosome distribution analysis indicated that a majority of the circRNAs were primarily located on chromosomes 1, 2, and 3 (**Figure 2C**). Differential expression analysis of the circRNAs was conducted using the edgeR v3.24.3 analytical model. Applying stringent screening criteria of |log_2_^FC^| > 1 and a P-value < 0.05, we identified 364 differentially expressed circRNAs. Specifically, 154 differentially expressed circRNAs were observed in JH and 142 in JL, compared to their respective controls at 0 h. Additionally, a comparative analysis between the two varieties at the same time point revealed 68 differentially expressed circRNAs in relation to JH (**Figure 2D**).

GO enrichment analysis of the parental genes for all differentially expressed circRNAs revealed significant involvement in various biological processes. These included cellular metabolic compound salvage, reductive pentose-phosphate cycle, photosynthesis (encompassing both dark reaction and photorespiration), carbon fixation, protein autophosphorylation, sulfur amino acid biosynthetic processes, and Golgi localization (**Supplementary Table 5**). Concurrently, KEGG enrichment analysis identified several significantly enriched pathways for these differentially expressed circRNAs. Notable pathways included glyoxylate and dicarboxylate metabolism, taurine and hypotaurine metabolism, carbon fixation in photosynthetic organisms, arachidonic acid metabolism, ubiquinone and other terpenoid-quinone biosynthesis, selenocompound metabolism, biosynthesis of secondary metabolites, isoquinoline alkaloid biosynthesis, and tropane, piperidine, and pyridine alkaloid biosynthesis (**Supplementary Table 6**) (**Figure 2E**).

### 3.5 Identification and differential analysis of miRNAs

**Figure 3:**
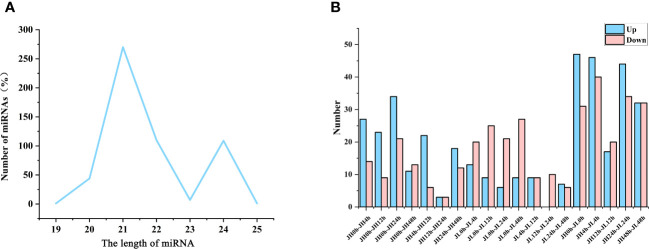
Identification and differential analysis of miRNAs in rice seedling leaves. **(A)** Length distribution of the identified miRNAs. **(B)** Count of differentially expressed genes in JH and JL varieties at various time points following cold treatment.

miRNA sequencing was performed on all samples, generating 409,055,593 clean reads. Using Bowtie software (version 1.1.2), we identified 542 miRNAs (**Supplementary Table 7**); 409 of these were previously known, while 113 were newly discovered. The majority of identified miRNAs had lengths of either 21 nucleotides (nt) (49.82% miRNAs) or 22 nt (20.30% miRNAs) (**Figure 3A**). Utilizing edgeR, we conducted a differential expression analysis of miRNAs based on the criteria of |log_1.5_^FC^| ≥ 1 and P ≤ 0.05. For the JH and JL varieties, differentially expressed miRNAs were identified at 0 h vs 4 h, 0 h vs 12 h, 0 h vs 24 h, and 0 h vs 48 h time points, with the counts being (41, 32, 55, 24) and (33, 34, 27, 36), respectively. A comparative analysis between JH and JL at these time points revealed the presence of 78, 86, 37, 78, 64 miRNAs that were differentially expressed in both varieties (**Figure 3B**).

### 3.6 Construction of ceRNA networks

**Figure 4:**
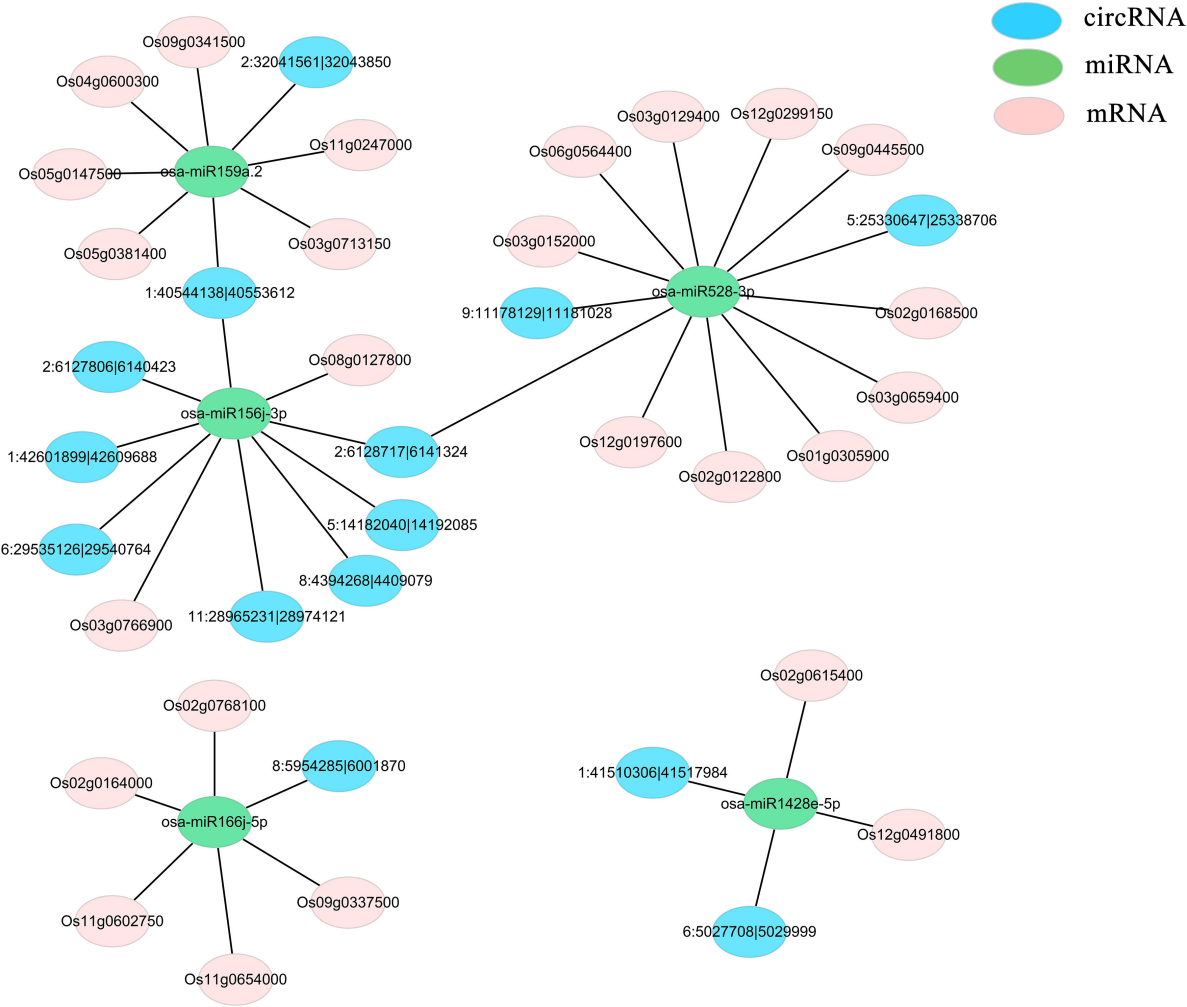
CeRNA network comprising significantly differentially expressed mRNAs, miRNAs, and circRNAs. Blue represents circRNAs, pink denotes mRNAs, and green indicates miRNAs.

The regulatory network involving circRNAs, miRNAs, and mRNAs functioning in response to low-temperature stress in rice was investigated. Differentially expressed miRNAs that appeared more than five times in each comparison were filtered and eventually the following five key miRNAs were identified: osa-miR528-3p, osa-miR166j-5p, osa-miR159a.2, osa-miR156j-3p, and osa-miR1428e-5p. Target genes for these miRNAs were predicted using psRNATarget software (Version2017) (**Supplementary Table 8**). Among the significantly differentially expressed mRNAs, those showing inverse expression patterns relative to the miRNAs were selected, yielding a total of 25 relevant target genes. The circRNA-miRNA interaction pairs were forecasted using TargetFinder (Version 2.0) software (**Supplementary Table 9**), and 16 differentially expressed circRNAs were identified using a similar methodology. For these predicted relationship pairs, RNAhybrid and psRobot software were used to conduct thermodynamic calculation of RNA structure formation for RNA : RNA interaction region analysis, further confirming the reliability of the results obtained by psRNATarget and TargetFinder software (**Supplementary Tables 10**-**13**). Subsequently, five ceRNA networks were established, elucidating the reciprocal relationships among these circRNAs, miRNAs, and mRNAs (**Supplementary Table 14**) (**Figure 4**).

### 3.7 Validation of differentially expressed genes

To validate our RNA-Seq findings, we performed qPCR analysis on selected candidate genes from the ceRNA network. We compared the log-fold changes for these 10 selected differentially expressed genes between the RNA-Seq and qRT-PCR datasets. Each sample for qRT-PCR was analyzed using three technical and three biological replicates. A correlation coefficient (R^2^) of 0.8795 was obtained (**Supplementary Figure 2**), demonstrating strong agreement between the qRT-PCR and RNA-Seq data. This result reinforces the reliability of our RNA-Seq findings.

### 3.8 Sequence variation analysis of *OsWRKY61*

**Figure 5:**
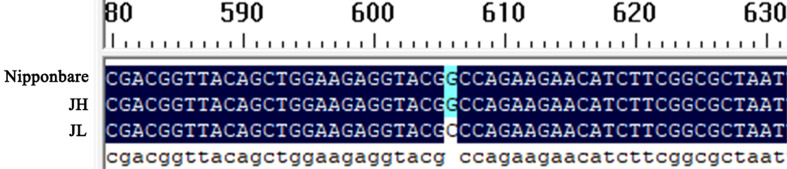
Comparative analysis of CDS differences in *OsWRKY61* between the two rice varieties. The Nipponbare genome serves as the reference sequence.

To further investigate the role of *OsWRKY61* in modulating cold tolerance in rice, we amplified the coding sequence (CDS) of *OsWRKY61* from JH and JL rice varieties. The PCR was carried out using the Nipponbare genome as a reference sequence, followed by separate sequencing of each variety. In comparison to the Nipponbare sequence, the CDS of JH was identical. However, an SNP was identified in the CDS sequence of JL when compared to the Nipponbare reference (**Figure 5**); specifically, where Nipponbare has an alanine at this base, JL features an arginine.

### 3.9 Phenotype identification of *OsWRKY61* overexpression plants

Initial DNA-level identification was performed on both T0 and T1 generations. Subsequent analysis focused on the T2 generation, where PCR and qRT-PCR methods were employed to evaluate plants with *OsWRKY61* overexpression. Firstly, DNA samples were extracted from *OsWRKY61* overexpression plants (labeled as WRKYOE-1 and WRKYOE-2) and the WT plants. These DNA samples served as templates for PCR amplification using expression vector primers, whose sequences are provided in **Supplementary Table 1**. No bands were detected in the WT samples, whereas distinct bands with consistent PCR product lengths were observed in the *OsWRKY61* overexpression plants (WRKYOE-1 and WRKYOE-2). This confirmed that the vector was absent in the WT plants. Next, RNA was extracted from both the *OsWRKY61* overexpression plants (WRKYOE-1 and WRKYOE-2) and the WT plants and reverse-transcribed into cDNA. This cDNA served as the template for the RT-qPCR reaction, carried out according to the previously established RT-qPCR protocol. The data indicated that the expression levels of *OsWRKY61* in WRKYOE-1 and WRKYOE-2 were significantly higher than those in the WT plants (**Supplementary Figure 3**). This provides evidence that *OsWRKY61* was overexpressed in the WRKYOE-1 and WRKYOE-2 plants.

**Figure 6:**
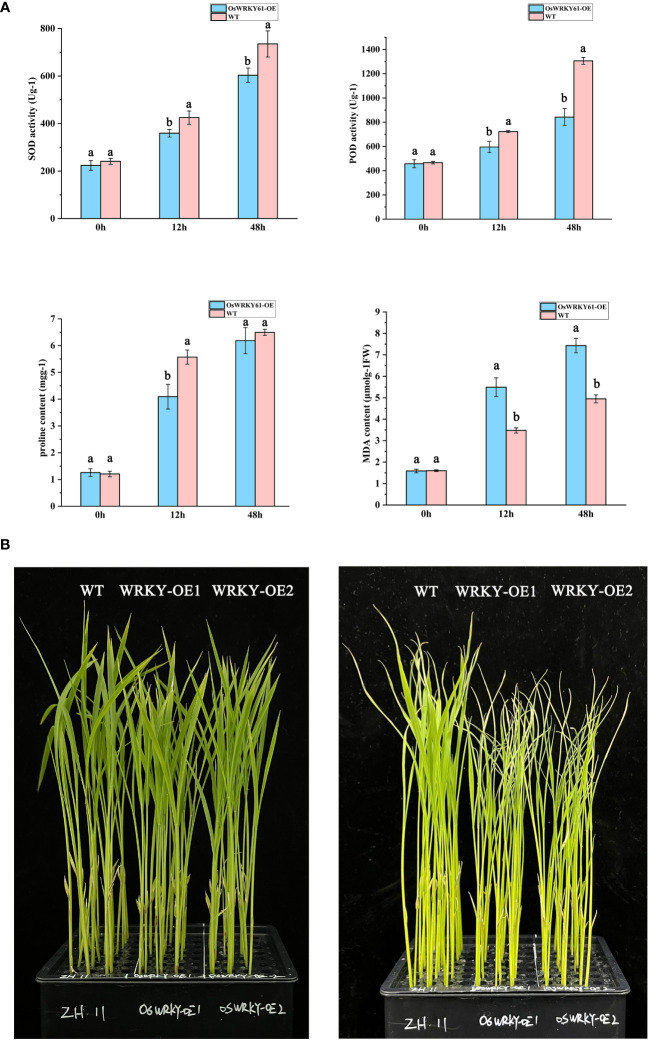
Physiological characterization of *OsWRKY61* overexpression plants following cold treatment. **(A)** Changes in SOD, POD, PRO, and MDA content levels in both overexpression and wild-type (WT) strains after low-temperature exposure. Labels “a” and “b” indicate statistical significance between varieties at the 0.05 level. **(B)** Phenotypic evaluation of overexpression and WT strains before and after both normothermic and low-temperature treatments. The left panel displays phenotypes before the initiation of low-temperature treatment, while the right panel shows phenotypes after 5 days of low-temperature exposure.

SOD, POD, PRO, and MDA are vital indicators for assessing cellular physiological responses to cold stress. After 12 and 48 h cold treatment, both wild-type and overexpression plants showed elevated levels of SOD, POD, PRO, and MDA compared to the levels of the control group at 0 h. While the PRO content was comparable between the wild-type and overexpression plants at 48 h, the overexpression plants demonstrated lower levels of SOD, POD, PRO, and MDA throughout the study compared to the wild-type plants (**Figure 6A**). The discrepancies in these physiological indicators between the wild-type and overexpression plants became more pronounced as the duration of cold treatment increased. Furthermore, the overexpression plants displayed heightened phenotypic vulnerability to low temperatures in comparison to their wild-type counterparts (**Figure 6B**). These results suggest that *OsWRKY61* overexpression reduces cold tolerance in rice seedlings.

## 4 Discussion

### 4.1 Identification and characterization of circRNAs in rice

circRNAs, a subclass of non-coding RNAs, have increasingly gained attention for their roles in various biological processes such as stress responses, growth, and development (Zhu et al., 2019). Advances in high-throughput sequencing and bioinformatics have enabled the identification of circRNAs associated with a range of stress conditions, including high temperatures (Zhao et al., 2020), salinity (Dong et al., 2022), and senescence (Huang et al., 2021). Nonetheless, the role of the ceRNA network in rice’s response to low-temperature stress remains largely unexplored.

In the current study, we identified a total of 3,387 circRNAs, consisting of 3,036 circRNAs in the cold-sensitive JH variety and 2,861 circRNAs in the cold-tolerant JL variety. A comparative analysis between the two varieties revealed that 2,510 circRNAs were expressed in both types (**Figure 2A**), emphasizing the unique, variety-specific expression patterns of circRNAs in rice seedlings.

### 4.2 Rice circRNAs and their role in low-temperature stress response

The response of rice to low-temperature stress is a multifaceted process, involving the activation of low-temperature-responsive genes and signaling pathways. In our study, we identified 364 circRNAs that exhibited significant differential expression under conditions of low-temperature stress (**Figure 2D**). Specifically, 154 and 142 differentially expressed circRNAs were identified in the cold-sensitive JH variety and the cold-tolerant JL variety, respectively, in comparison to the control group at 0 h. Additionally, a side-by-side comparison of the two varieties at the same time point revealed 68 differentially expressed circRNAs in JH relative to those in JL. GO and KEGG pathway enrichment analyses of the host genes for these differentially expressed circRNAs indicated their involvement in a diverse set of metabolic pathways. Notably, these host genes were enriched in pathways related to cellular metabolic compound salvage, the reductive pentose-phosphate cycle, various aspects of photosynthesis (including dark reactions and photorespiration), carbon fixation, protein autophosphorylation, glyoxylate and dicarboxylate metabolism, carbon fixation in photosynthetic organisms, arachidonic acid metabolism, and terpenoid-quinone biosynthesis (**Figure 2E**). Significantly, enhanced activity in the pentose-phosphate cycle has been linked to increased NADPH content, which is known to improve a plant’s ability to neutralize ROS and thereby enhance cold tolerance (Tian et al., 2021).

Protein phosphorylation pathways play critical roles in responses to various stressors (Tian et al., 2021). A proteomic analysis of winter turnip rape under cold stress revealed significant enrichment in similar metabolic pathways, including those involved in photosynthesis and carbon metabolism (Xu et al., 2018). Consistently, our study also identified a significant number of pathways related to photosynthesis and carbon metabolism, such as photosynthesis, dark reaction, photorespiration, carbon fixation, glyoxylate and dicarboxylate metabolism, and carbon fixation in photosynthetic organisms. This leads us to hypothesize that low-temperature conditions have a considerable impact on photosynthesis and carbon metabolism in plants, which will be a key area for our future research endeavors.

### 4.3 Potential circRNA-miRNA-mRNA regulatory network in rice in response to low-temperature stress

circRNAs modulate the expression of miRNA target genes by competitively binding to miRNAs, thereby suppressing latter’s silencing effect on these target genes. In this study, we investigated the circRNA-miRNA-mRNA regulatory network relevant to low-temperature stress in rice. We constructed five circRNA-miRNA-mRNA regulatory modules potentially associated with rice’s response to such stress conditions (**Figure 4**). By examining the functions of these differentially expressed target genes, we aim to elucidate the potential roles of these circRNA-miRNA-mRNA regulatory modules. In previous research, osa-miR528-3p was found to be significantly upregulated under cadmium stress in two rice varieties (Liu et al., 2020). However, our present study revealed that this gene was markedly downregulated during 4, 12, 24, and 48 h cold treatment in the JL variety, as well as at 4, 12, and 24 h when comparing the JL variety to the JH variety. miR166 enhances the resistance to abiotic stress in rice by mitigating oxidative damage. Additionally, miR166k and miR166h are implicated in regulating disease resistance in rice (Ding et al., 2018; Salvador-Guirao et al., 2018; Zhang et al., 2018). Furthermore, members of the miR166 family, including osa-miR166h-3p, osa-miR166g-3p, osa-miR166j-5p, osa-miR166k-3p, and osa-miR166l-3p, were differentially expressed in rice variety 9311 under low-temperature stress (Zhao et al., 2022).

In this study, osa-miR166j-5p exhibited significant downregulation at 4, 12, 24, and 48 h of cold treatment in the cold-tolerant JL variety. It was also significantly upregulated at 48 h compared to its expression in the cold-sensitive JH variety. These findings further underscore osa-miR166j-5p’s critical role in regulating cold tolerance in rice. In a separate study on salt-treated rice seedlings, osa-miR156j-3p was notably downregulated (Goswami et al., 2017). Osa-miR156 is implicated in regulating multiple abiotic stress responses and demonstrates contrasting expression patterns when exposed to different abiotic stressors. For example, while osa-miR156 exhibited increased expression upon radiation exposure, it showed the opposite trend under low-temperature or drought conditions (Zhang et al., 2013).

In our study, this gene was notably upregulated at 4, 12, 24, and 48 h of cold treatment in the JH variety. This suggests that osa-miR156 serves as a stress regulator with distinct expression behaviors under different stress conditions. Among the 25 differentially expressed mRNAs, *Os03g0152000*, which is part of the antioxidant system, showed significant upregulation in the cold-tolerant JL variety at 4, 12, 24, and 48 h of cold treatment. This gene features a structural domain encoding a redox-active, copper-linked protein. This domain is also found in enzymes like laccase, SOD, and multi-copper oxidase, which catalyze a variety of cellular processes. Previous research (da Maia et al., 2016) also demonstrated that this gene was upregulated in cold-tolerant (Oro) varieties and downregulated in cold-sensitive (Tio Taka) varieties. *Os12g0491800*, previously implicated in diterpenoid biosynthesis studies on low-temperature tolerance in rice, exhibited differential expression according to both transcriptomic and proteomic data. When compared to the JH variety, this gene was notably downregulated at 4, 12, 24, and 48 h. *Os05g0381400*, known to respond to heat stress in rice, was significantly downregulated at the 24-hour mark in comparison with the JH variety in this study. This gene may be responsive to both low and high temperature stress conditions in rice. Collectively, these genes are promising candidate genes for stress responses in rice and offer valuable reference points. Our findings lay the groundwork for further exploration of the circRNA-miRNA-mRNA regulatory network in rice under low-temperature stress conditions.

The prediction of competing endogenous RNA (ceRNA) networks, while a significant advancement in understanding RNA interactions and gene regulation, comes with limitations that necessitate additional bioinformatic and experimental work for validation caused by complex post-transcriptional regulation mechanisms. Computational models often rely on simplified assumptions about RNA interactions, which may not fully capture the intricacies of miRNA binding and the nuances of molecular competition. Additionally, the transient and context-specific nature of ceRNA interactions makes it difficult to generalize findings across different cell types or physiological conditions. To address these limitations, experimental validation is equally essential to confirm ceRNA predictions. Techniques like luciferase reporter assays, RNA immunoprecipitation, and gene knockdown or overexpression studies can provide direct evidence of ceRNA interactions and their functional consequences. In summary, ceRNA network prediction requires a combination of sophisticated bioinformatic modeling and rigorous experimental validation to overcome its inherent limitations and prove the existence and functional significance of the predicted ceRNA candidates.

### 4.4 Functional validation of *OsWRKY61*

We subsequently analyzed the CDS of *OsWRKY61* and found that its sequence in the cold-sensitive JH variety was consistent with that in the Nipponbare variety. In contrast, the cold-tolerant JL variety had one SNP in the CDS compared to Nipponbare (**Figure 5**). This single-base variation, resulting in a non-synonymous amino acid substitution, may account for the significant difference in cold tolerance between the two varieties. To explore this, we conducted physiological and phenotypic assessments of *OsWRKY61*-overexpressing plants under low-temperature stress. Our observations revealed that the overexpression plants were notably less cold-tolerant than their wild-type counterparts (**Figure 6B**), shedding light on the role of this particular transcription factor in cold tolerance.

*OsWRKY71* transgenic rice lines, OX12 and OX21, have demonstrated superior cold tolerance in terms of survival rate, photosynthetic capacity, fresh weight, and dry weight when subjected to cold treatment at 4°C (Kim et al., 2016). Under normal conditions, the OsWRKY63 overexpression strain showed no significant differences compared to the wild type. However, under cold stress, the strain had a significantly reduced fruit set rate and lower seedling cold-stress survival than the wild type. Intriguingly, the use of CRISPR/Cas9 technology to knock down OsWRKY63 expression led to enhanced cold tolerance (Zhang et al., 2022). Similarly, OsWRKY45-1 and OsWRKY45-2 have been found to negatively regulate cold tolerance in rice; overexpressing these genes resulted in a markedly lower survival rate after cold treatment compared to wild-type plants (Tao et al., 2011; Zhang et al., 2021). While these findings provide valuable insights, additional research is needed to confirm these conclusions. Future work should focus on the construction of *OsWRKY61* mutants and the use of RNA interference techniques to further clarify the role of this candidate gene in the signaling pathways associated with low-temperature stress.

## Data availability statement

The datasets presented in this study can be found in online repositories. The names of the repository/repositories and accession number(s) can be found below: Bioproject accession number: PRJNA1071886 and PRJNA1068025.

## Author contributions

HW: Writing – original draft, Writing – review & editing. YJ: Resources, Funding acquisition, Writing – original draft. XB: Writing – original draft, Data curation. JW: Writing – original draft, Conceptualization. GL: Writing – original draft, Formal analysis. HXW: Writing – original draft, Methodology. YW: Writing – original draft, Project administration. JX: Writing – original draft, Software. HM: Writing – original draft, Supervision. ZL: Writing – original draft, Validation. DZ: Visualization, Writing – review & editing. HZ: Resources, Funding acquisition, Writing – original draft, Writing – review & editing.

## Supplementary material

Supplementary Figure 1 Logarithmic values of identified circRNAFPKM.

Supplementary Figure 2 Correlation coefficient (R^2^) plots comparing differential gene expression data from RNA-Seq and qRT-PCR analyses.

Supplementary Figure 3 Construction and molecular characterization of *OsWRKY61* overexpression plants. **(A)** PCR-based identification of WT along with OsWRKYOE-1 and OsWRKYOE-2 overexpression plants at the DNA level. Labels “a” and “b” indicate statistical significance between varieties at the 0.05 level. **(B)** RNA-level characterization using RT-qPCR for WT, OsWRKYOE-1, and OsWRKYOE-2.
