# Supplementary figures and images for: Whole-transcriptome profiling and identification of cold tolerance-related ceRNA networks in japonica rice varieties
Source: Front Plant Sci. 2024 Mar 19;15:1260591. doi: 10.3389/fpls.2024.1260591 (PMC10985246; doi:10.3389/fpls.2024.1260591)

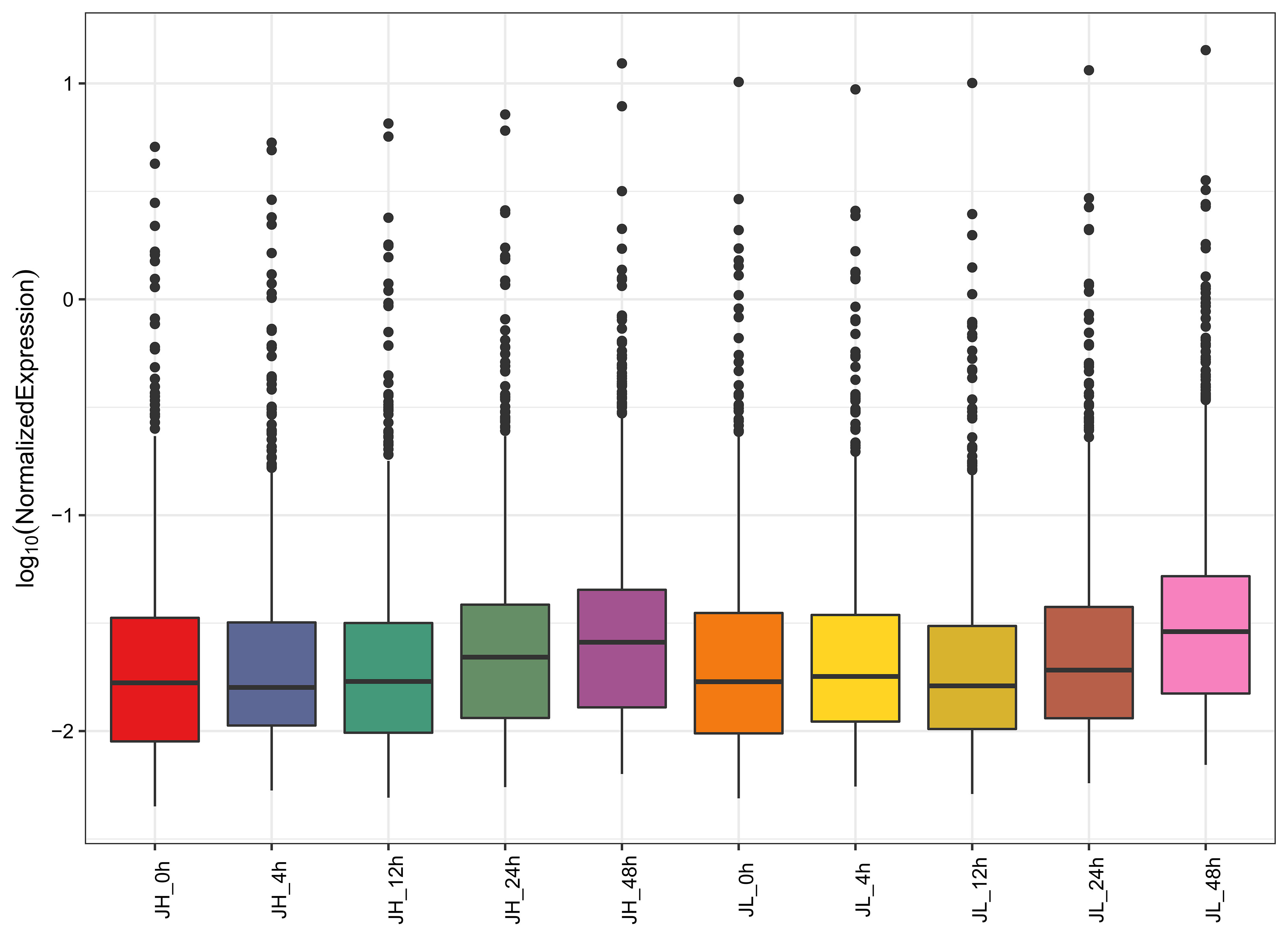

Supplement: Supplementary Figure 1 — Logarithmic values of identified circRNAFPKM. [file Image_1.jpeg]

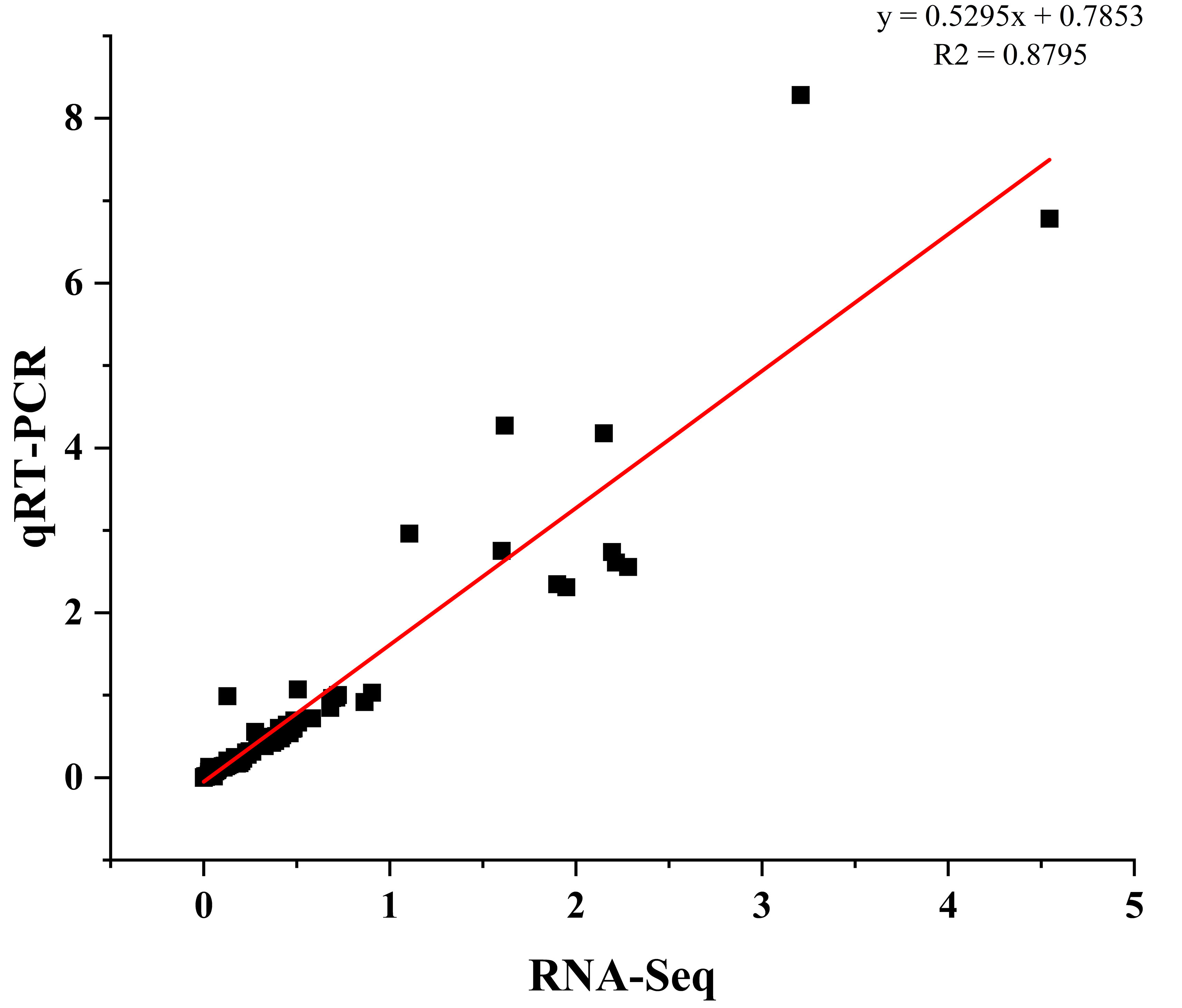

Supplement: Supplementary Figure 2 — Correlation coefficient (R2) plots comparing differential gene expression data from RNA-Seq and qRT-PCR analyses. [file Image_2.jpeg]
